# Design of Jitter Compensation Algorithm for Robot Vision Based on Optical Flow and Kalman Filter

**DOI:** 10.1155/2014/130806

**Published:** 2014-01-29

**Authors:** B. R. Wang, Y. L. Jin, D. L. Shao, Y. Xu

**Affiliations:** ^1^College of Mechanical and Electrical Engineering, China Jiliang University, Hangzhou, Zhejiang 310018, China; ^2^Department of Control Science and Engineering, Zhejiang University, Hangzhou, Zhejiang 310007, China

## Abstract

Image jitters occur in the video of the autonomous robot moving on bricks road, which will reduce robot operation precision based on vision. In order to compensate the image jitters, the affine transformation kinematics were established for obtaining the six image motion parameters. The feature point pair detecting method was designed based on Eigen-value of the feature windows gradient matrix, and the motion parameters equation was solved using the least square method and the matching point pairs got based on the optical flow. The condition number of coefficient matrix was proposed to quantificationally analyse the effect of matching errors on parameters solving errors. Kalman filter was adopted to smooth image motion parameters. Computing cases show that more point pairs are beneficial for getting more precise motion parameters. The integrated jitters compensation software was developed with feature points detecting in subwindow. And practical experiments were conducted on two mobile robots. Results show that the compensation costing time is less than frame sample time and Kalman filter is valid for robot vision jitters compensation.

## 1. Introduction

Computer vision is the most important sensor of intelligent moving robots. In real environment, the surface evenness always causes the camera jitters to affect the precision of operation. Electronic vision stabilization has been widely used in the autonomous robot vision [1–4], which includes motion estimation, smoothing and compensation processes [5]. The method of BMA (Blocks Matching Algorithm) was provided with the exhaustive search method in [6]. BMA can get high precision with large amount of calculation. However, its real time capability is bad and just fit for the simple motion vision [7]. Reference [8] used the circular block matching to improve the estimation for the rotational motion vision. Reference [9] researched a matching algorithm based on splitting and merging of block and representative points to improve the calculation speed. Projection algorithm (PA) was put forward for the vision motion estimation to gain the displacement vector based on the grayscale change of images. However, PA is valid for these images with the obvious grayscale change. Reference [10] researched the feature-tracking algorithm (FTA). Reference [11] used the optical flow constraints equations to solve the motion parameters of images. Optical flow is the 2D instantaneous velocity field of moving points in focal plane array. The aim of motion error compensation is to reconstruct images based on the smoothed parameters. The mean filter was used for smoothing motion vector in [12]. Reference [13] put motion parameters into the finite impulse response (FIR) filter and filtered them with product of input sequences and interruptive function. To distinguish the independent movement from the jitters, Kalman filter was used in image stabilization [14]. Kalman algorithm can predict the image motion and adjust observation data based on error covariance. Moreover, authors had compared the translation jitters to rotation jitters, FIR filter to Kalman filter, and relative parameters filter to absolute parameters filter, and the advantages and disadvantages of various algorithms were given out clearly [15]. From the above literatures, we found that the analysis of images motion equations solving error is less, especially in view of feature point pairs number and matching error, and jitters compensation test is absent on the autonomous robot moving on bricks road in outroom.

The rest of this paper is organized as follows. Firstly, an image kinematics model is established and the feature points detecting and matching methods are designed based on the gradient matrix Eigen-value and the optical flow, and image motion parameters solving method is given in Section 2. A jitters compensation process based on filter is described in Section 3. Then the condition number of the equation coefficient is used to analyse the parameters error. A compensation software is developed and experiments are implemented based on the platform of two autonomous robots moving in outroom in Section 4, and curves are compared between before and after Kalman filter. Finally, results are given in Section 5.

## 2. Kinematics Modelling and Solving

### 2.1. Kinematics Modelling of Images

Coordination of the pixel *P* is defined as *P*(*u*, *v*, *t*) at time *t* in the image coordinate system. (*u*_1_, *v*_1_) denotes the coordination of *P* in the given frame and (*u*_2_, *v*_2_) in the adjacent frame. According to the imaging producing principle, *P* coordination moving equations are described as

(1) u2=z1z1+Δz(u1cos⁡θ+v1cos⁡θ) +f(−Δxcos⁡θ−Δysinθ)z1+Δz,

(2) v2=z1z1+Δz(−u1sin⁡θ+v1cos⁡⁡θ) +f(Δxsin⁡θ−Δycos⁡⁡θ)z1+Δz,

where *f* is the focal length of camera and *z*_1_ is the position of *P* in direction of optical axis of camera coordinate system. Δ*x*, Δ*y*, and Δ*z* are the amount of coordination increment, and *θ* is the rotation amount. According to (1), images motion is relative to 6 parameters. So the image motion kinematics can be established as

(3) $$\begin{array}{c} \left[\begin{array}{c} u_{2}\\ v_{2}\\ 1 \end{array}\right]=A\left[\begin{array}{c} u_{1}\\ v_{1}\\ 1 \end{array}\right]=\left[\begin{array}{ccc} a_{0} & a_{1} & a_{2}\\ a_{3} & a_{4} & a_{5}\\ 0 & 0 & 1 \end{array}\right]\left[\begin{array}{c} u_{1}\\ v_{1}\\ 1 \end{array}\right], \end{array}$$

where *a*_0_, *a*_1_, *a*_3_, and *a*_4_ indicate the scale and rotation amount, and *a*_2_ and *a*_5_ the translation amount.

### 2.2. Feature Point Detecting

The feature window is defined as a *j* × *j* (*j* is odd number) square, and the center point of this feature window is, namely, the feature point. Gradient matrix of the feature window is

(4) $$\begin{array}{c} G=\left[\begin{array}{cc} \sum_{w}^{}g_{x}^{2} & \sum_{w}^{}g_{x}g_{y}\\ \sum_{w}^{}g_{x}g_{y} & \sum_{w}^{}g_{y}^{2} \end{array}\right], \end{array}$$

where *w* represents the feature window scope and *g*_*x*_ and *g*_*y*_ represent the gradients in horizontal and vertical direction, respectively, which can be got by numerical difference.

Using  *k* × *k*  (*k* < *j*) subwindow to scan the feature window along horizontal and vertical direction, we can get (*j* − *k*)^2^ scanning windows. Each gradient matrix of the scanning window has two real Eigen-values, and the lesser Eigen-value is denoted by EV_min⁡_. The maximum value of the lesser Eigen-value of all scanning windows can be expressed as

(5) EV=max⁡{EV1min⁡,EV2min⁡,…,EVimin⁡}i=1,2,…,(j−k)2.

This paper adopts EV to describe the center point characteristics quantity. If EV is larger than the given threshold, this point will be selected as the useful feature point.

### 2.3. Feature Points Matching Based on Optical Flow

When robot is moving in the continuous surface, the adjacent points have homothetic motions, constant brightness, and a tiny small motion in continuous time.

The frames constraint equation can be transformed using Taylor formula [11]; the following equation can be got

(6) $$\begin{array}{c} I_{x}u^{\prime }+I_{y}v^{\prime }+I_{t}=0, \end{array}$$

where *I*_*x*_ = ∂*I*/∂*x* and *I*_*y*_ = ∂*I*/∂*y* are the derivatives in horizontal and vertical direction, respectively, *I*_*t*_ = ∂*I*/∂*t* is the derivative of time, and *u*′ and *v*′ are the coordination difference of the feature point, namely, the gradient of optical flow.

We establish (6) of all points in 5 × 5 square and use the least square method to solve the *u*′ and *v*′. Based on the *u*′ and *v*′, the feature point's corresponding pixels coordinate in adjacent frame can be got.

### 2.4. Kinematics Parameters Solving

The aim of kinematics parameters solving is to get the image motion parameters *a*_0_, *a*_1_, *a*_2_, *a*_3_, *a*_4_, and *a*_5_. According to (3), there are 6 parameters needing to be solved, so at least 3 pairs of matching points are necessary. To ensure precise solving and stability, the number of pairs, denoted by *K*, is always more than 3. The solving equation of the kinematics parameters is established as

(7) $$\begin{array}{c} \left[\begin{array}{c} a_{0}\\ a_{1}\\ a_{2}\\ a_{3}\\ a_{4}\\ a_{5} \end{array}\right]={\left[\begin{array}{cccccc} u_{1} & v_{1} & 1 & 0 & 0 & 0\\ 0 & 0 & 0 & u_{1} & v_{1} & 1\\ u_{2} & v_{2} & 1 & 0 & 0 & 0\\ 0 & 0 & 0 & u_{2} & v_{2} & 1\\ \vdots & \vdots & \vdots & \vdots & \vdots & \vdots \\ u_{K} & y_{K} & 1 & 0 & 0 & 0\\ 0 & 0 & 0 & u_{K} & v_{K} & 1 \end{array}\right]}^{-1}\left[\begin{array}{c} u_{1}^{\prime }\\ v_{1}^{\prime }\\ u_{2}^{\prime }\\ v_{2}^{\prime }\\ \vdots \\ u_{K}^{\prime }\\ v_{K}^{\prime } \end{array}\right]=B\left[\begin{array}{c} u_{1}^{\prime }\\ v_{1}^{\prime }\\ u_{2}^{\prime }\\ v_{2}^{\prime }\\ \vdots \\ u_{K}^{\prime }\\ v_{K}^{\prime } \end{array}\right], \end{array}$$

where (*u*_*i*_, *v*_*i*_) and (*u*_*i*_′, *v*_*i*_′) are the matching point coordinate position of the feature point, respectively.

When *K* > 3, (7) is an overdetermined equation and can also be solved using the least square method.

## 3. Jitter Compensation and Errors Analysis

Using *q*_*n*−*m*_ and *q*_*n*_ to denote the corresponding pixels coordinates in the frames, of *I*_*n*−*m*_ and *I*_*n*_ separated by *m* frames respectively, we can transform (3) as the affine transformation kinematics model through the recursive method

(8) $$\begin{array}{c} q_{n}=F_{n}^{n-m}q_{n-m}+D_{n}^{n-m}, \end{array}$$

where *m* = 1,2,…, *n* − 1, *F*_*n*_^*n*−*m*^ = ∏_*k*−1_^*m*^ *F*_*n*−*k*+1_^*n*−*k*^, *D*_*n*_^*n*−*m*^ = ∑_*k*−1_^*m*−1^(∏_*l*−1_^*k*^ *F*_*n*−*l*+1_^*n*−*l*^)*D*_*n*−*k*_^*n*−*k*−1^ + *D*_*n*_^*n*−1^, $F_{n}^{n-1}=\left[\begin{array}{cc} a_{0} & a_{1}\\ a_{3} & a_{4} \end{array}\right]$, and $D_{n}^{n-1}=\left[\begin{array}{c} a_{2}\\ a_{5} \end{array}\right]$, *n* = 2, 3, …, *N*_*f*_, and where *N*_*f*_ is the largest frame count of the video.

According to the coordinate relations of the matching feature points, *F* and *D* can be got through the same solving method as (7).

Due to the jitters, the motion parameters ${\left[\begin{array}{cccccc} a_{0} & a_{1} & a_{2} & a_{3} & a_{4} & a_{5} \end{array}\right]}^{T}$ are not smooth. This paper used Kalman filter to smooth the parameters and then get the new *F* and *D*.

In motion compensation process, (8) was solved again with the new *F* and *D* to get the smoother *q*_*n*_ and *q*_*n*−*m*_. Whole continuous and post compensation stable video can be obtained using the first frame image and recursion algorithm.

The motion parameters are the basics of compensation, so the parameters solving errors will obviously affect jitters compensation. For the purpose of errors analysis, we adopted the condition number of matrix to quantificationally illustrate effect of the feature points quantity on parameters solving errors.

In mathematics, the matrix condition number is defined as the product of matrix norm and its inverse matrix norm and expresses the sensitivity of matrix calculation to error.

The condition number of the matrix *B* of (7) was given by

(9) $$\begin{array}{c} \text{cond}\left(B\right)=\left|B\right|\cdot \left|B^{-1}\right|, \end{array}$$

where |·| is a symbol of matrix norm.

Following analysis is about two solution stages of the compensation: images motion kinematics parameters solving using (7) and jitters compensation using (3).

In view of (7), if the matrix *B* has a big condition number, the slim errors of feature point matching will cause a huge change on kinematics parameters. In order to analyse the effect of the numbers of the feature points on the solve precision, two computing examples were carried out.

Firstly, 3 pairs of the feature points, (39,29)→(39,32), (81,22)→(81,25), and (102,36)→(102,39), were used for solving (7).

Then designedly add 1 pixel error in vertical direction to the 3rd feature matching points, such as (102,36)→(102,40), and (7) was solved again.

Similarly, 30 pairs of the feature point, including the above 3 pairs, were used for solving (7), and also designedly add 1 pixel error to the 3rd point. Kinematics parameters solving results are shown in Table 1. The bold numbers in Table 1 refer to the parameters with errors. Table 1 shows that solving with 30 pairs matching points is robust to the matching error, and the individual point matching error would not produce a large change on results. The reason is that the condition number of *B* with 30 pairs is much lesser than with 3 pairs. So the solving method just using 3 pairs matching points is more sensitive to the matching error.

In view of (3), using parameters got by (7) to compute the condition number of *A*, solve the corresponding point of the compensation processing. Results are shown in Table 2. The bold numbers in Table 2 refer to the parameters with errors.

Table 2 also shows that solving (3) with parameters got by 30 pairs matching points is robust and can get more precise results.

## 4. Vision Stabilization Software Testing

### 4.1. Test Setup

**Figure 1:**
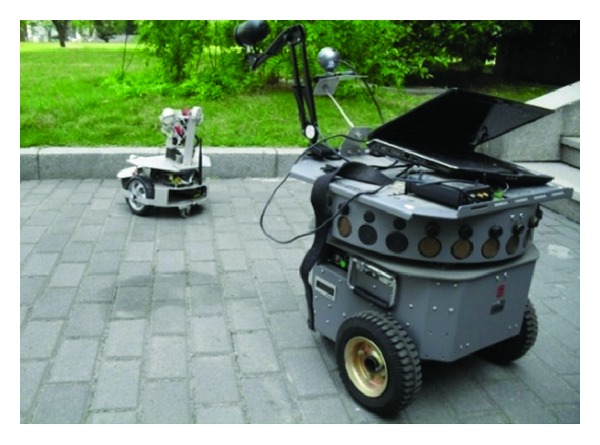
Two mobile robots used for experiments.

Experiments were conducted on two autonomous moving robots. Robot.1 (large) is the Voyager-IIA autonomous robot made in China. Robot.2 (small) is the X80-H robot made in Canada. Robot.1 has many sensors such as vision camera, ultrasonic, infrared ray, and gyroscope. Robot.2 is equipped with wireless communication equipment. The physical experiment scene is shown in Figure 1. In experiment processing of Robot.1 following Robot.2, the moving speed of Robot.1 is set to 0.11 m/s, while of Robot.2 to 0.07 m/s and linear forward.

The two autonomous moving robots are controlled by the personal computer (PC) through wireless network. Autonomous navigation software on PC controls motion of the autonomous mobile robot, such as move forward, turn back, speed up, and slow down. The CMOS camera is fixed on Robot.1 and connected with PC by USB line, and it transfers the real-time images to PC.

### 4.2. Jitter Compensation Software Design

**Figure 2:**
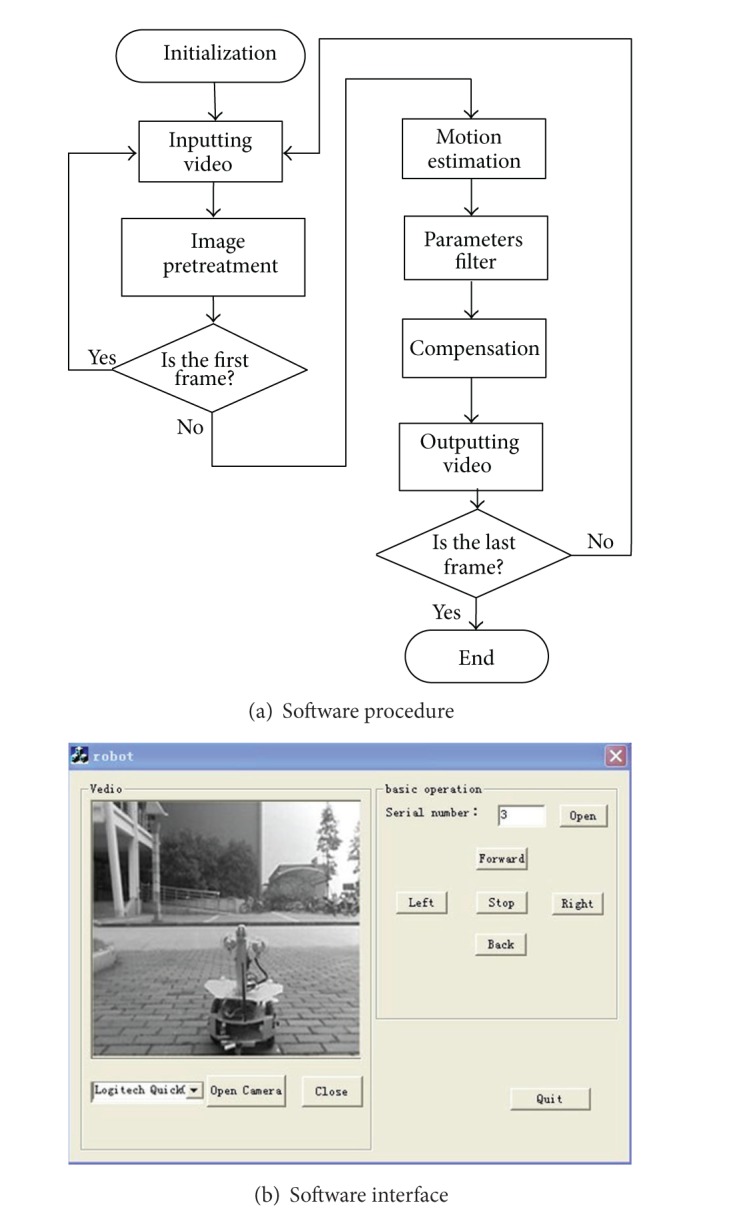
The compensation software.

Software was developed using the Visual C++6.0 programming language on the Windows XP operating system. And the central processing unit (CPU) is an Intel Core2Duo 2 GHz system with 1 GB of RAM. The whole software is composed of three parts, the control software of Robot.1, the control software of Robot.2, and the jitters compensation software. Video was captured based on DirectShow. After the image stabilization, the smooth video is displayed on the screen of  PC. The compensation software procedure is illustrated in Figure 2(a) and software visual interface in Figure 2(b).

The video sampling frequency in the mobile robot moving is 20 Hz; namely, the time interval of the adjacent frame is 50 ms. All jitters compensation time was tested through GetTickCount() and cvGetTickFrequency() functions provided by LIB files. And test result is about 24 ms, greatly less than 50 ms. So the proposed jitters compensation algorithm is real-time.

### 4.3. Subwindow Feature Point Detecting Experiment

**Figure 3:**
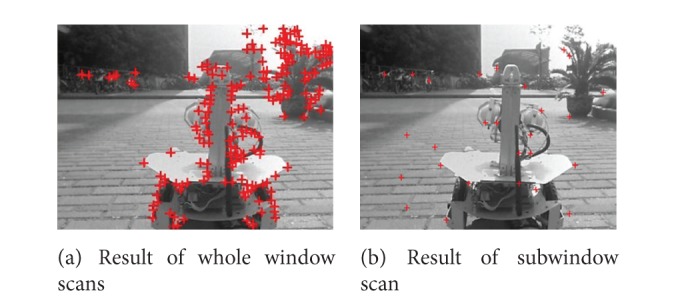
The comparisons of feature points detecting in whole window and subwindow.

The feature point detecting algorithm based on the gradient matrix Eigen-value always gets the feature points collected on the some objects. In order to uniformly distribute the feature points and accelerate the detecting speed, we divided whole image into many nonoverlapping domains with *s* × *s* square size. Then we scanned *s* × *s* subwindow to get feature points.  Figure 3 shows the feature points detecting of one frame in the video sequence based on the conventional feature extraction and the improved feature extraction.

Feature points may concentrate on some objects in Figure 3(a), such as Robot.2 and the tree in background, which will easily make wrong matching and is disadvantageous to the parameters solving.  Figure 3(b) shows that scan in subwindow makes the feature points equably distributed in the whole image. And the dispersed feature points are beneficial for the kinematics parameters solving using the least square method.

### 4.4. The Parameters Smooth Results Using Kalman Filter

Two robots moved linearly forward, respectively, apart by about 1.5 m. There is the same size blocks paved on robot moving road. The length and width of blocks are 19 cm and 9.4 cm, respectively, and slot between blocks is of 0.7 cm width and depth 0.3 cm. Robot.2 is forward, while Robot.1 is behind and its motion velocity is more than that of Robot.2. So Robot.1 is continuously getting closer to Robot.2. The test time of jitters compensation is 16 s.

**Figure 4:**
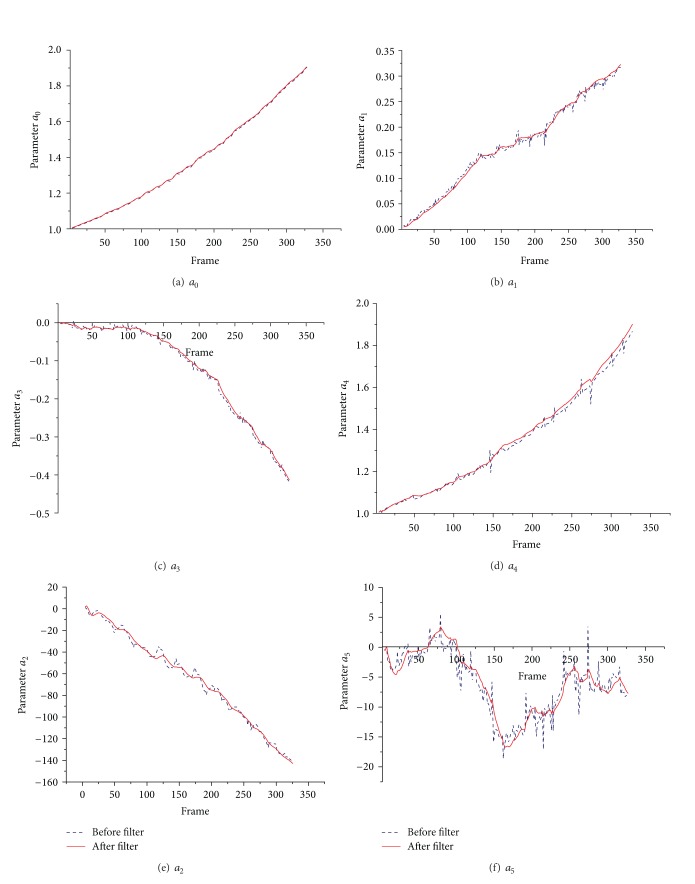
The filter results of matrix *A* parameters.

The process state variance of Kalman filter has key effect on parameters smooth degree of intended movement. Meanwhile, the observed variance of Kalman filter decides the changeability of unintended jitters movement. If observed variance is zero, it will cause no motion compensation effect. So the process state variance and observed variance values must be set according to intended movement and jitters motion quantity, respectively. The initial process state variance in Kalman filter is ${\left[\begin{array}{cccccc} 1 & 0 & 0 & 1 & 0 & 0 \end{array}\right]}^{T}$, the state square error is 0.00001, and the observed variance is 0.01. Filter was implemented with relative motion parameters of matrix *A*. Then, in order to show motion parameters clearly, the corresponding absolute parameters were got by successively adding relative parameters and are illustrated in Figure 4.

Mean square errors (MSE) comparisons between before and after filter are shown in Table 3.

Figure 4 shows that the curves after filter are smoother than before filter. The *a*_5_ variation is more complicated than other parameters. The reason is that the video is obtained during the mobile robot is moving on the road paved with bricks, and the interval slots between bricks mainly produce vibration of robot wheel in vertical direction. Table 3 shows the mean square error after filter is less than before evidently.

### 4.5. The Effect of Video Stabilization

**Figure 5:**
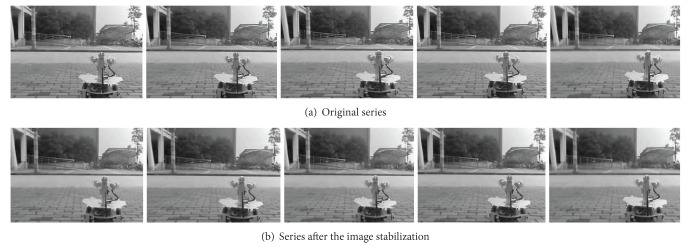
The comparisons of before and after the image stabilization.

The series frames of before and after the images stabilization are as shown in Figure 5.

Figure 5 shows the frames 132, 136, 140, 144, and 148 in turn. With Robot.1 moving, the left corner blacker brick should be continuously near to the bottom line of images. According to comparison of 132th frame to 136th frame in Figure 5(a), the blacker brick is almost motionless. According to comparison of 140th frame to 144th frame in Figure 5(a), the blacker brick is away from the bottom line instead. It is caused by the jitters of the robot wheels moving on bricks seam. And after image stabilization, the video sequence is clearly reflecting the motion of the blacker brick smoothly close to the bottom line of images.

Moving on the bricks seam will cause the video bidirectional shake, so Figure 5(a) shows the left-up corner aslant pillar is around swing. But this swing is slight in the video sequence after the image stabilization in Figure 5(b).

## 5. Conclusions

Based on comparative analysis, the following can be got.

1. The number of feature point pairs has great effect on the parameters solving precision, and this effect can be quantificationally analyzed by the condition number of matrices *A* and *B*. The condition number of *B* is far larger than *A*. Equation (7) is very sensitive to errors; kinematics parameters must be solved using the feature point pairs as many as possible to reduce the solving errors.
2. Subwindow feature point detecting can avoid the feature points gathering on some objects.
3. The visual jitters compensation algorithm based on optical flow and Kalman filter, developed based on PC, USB camera, Microsoft Windows operating system, and VC++, meets the requirements of precision and real-time demand of robot vision.

But the proposed method cannot compensate the migration jitters caused during the exposure time of the camera. Further study will focus on how to make the parameters of Kalman filter adaptively change with the different jitters amplitude and frequency.

## Figures and Tables

**Table 1:** Kinematics parameters errors analysis of 3 to 30 pairs feature points.

	*B* condition number	Kinematics parameters	Max. errors	Ideal kinematics parameters
3 pairs	1563.2	(1, 0, 0, **0.0095**, **1.0571**, **0.9714**)	2.0286	(1, 0, 0, 0, 1, 3)
30 pairs	347.5	(1, 0, 0, **0.0054**, **0.9981**, **3.2713**)	0.2713	

The bold numbers refer to the parameters with errors.

**Table 2:** Corresponding point errors analysis of 3 to 30 pairs points.

	*A* condition number	Corresponding point	Max. errors	Given point	Ideal corresponding point
3 pairs	13.5	(102, **319.01**)	16.01	(102, 300)	(102, 303)
30 pairs	12.6	(102, **303.25**)	0.25		

The bold numbers refer to the parameters with errors.

**Table 3:** The comparisons of matrix *A* parameters of before and after filter.

	*a*_0_	*a*_1_	*a*_3_	*a*_4_	*a*_2_	*a*_5_
Before	0.001434	0.004646	0.003686	0.010688	1.525818	0.348722
After	0.000711	0.000649	0.000874	0.001435	0.348722	0.267184
